# Innovative Detection and Mitigation of Ergot Alkaloids in Cereals: Advancing Food Safety

**DOI:** 10.3390/metabo15120778

**Published:** 2025-12-03

**Authors:** Maria Balatsou, Aikaterini Koutsaviti, Yiannis Sarigiannis, Christos C. Petrou

**Affiliations:** 1 Department of Health Sciences, School of Life & Health Sciences, University of Nicosia, 2417 Nicosia, Cyprus; balatsou.m1@live.unic.ac.cy (M.B.); koutsaviti.a@unic.ac.cy (A.K.); sarigiannis.i@unic.ac.cy (Y.S.); 2 Bioactive Molecules Research Center, University of Nicosia, 2417 Nicosia, Cyprus

**Keywords:** mycotoxins, ergot alkaloids, *Claviceps purpurea*, food safety, ergot alkaloids in food, blockchain food safety, machine learning ergot alkaloids

## Abstract

**Background/Objectives**: Ergot alkaloids are mycotoxins produced mainly by fungi of the genus *Claviceps*, infecting a wide variety of plants, especially cereals. These toxins usually manifest as black, hardened sclerotia (ergots), though they may also be invisible when dispersed in grain. They pose a significant risk to animals and humans when present in contaminated cereals. They can cause ergotism, with vasoconstriction, ischemia, hallucinations, and in severe cases gangrene. This study was carried out in response to the European legislative actions which determine the permissible levels of ergot alkaloids in cereals. Historically, consumers manually removed visible sclerotia from grain, and farmers applied fertilizers or timed harvests to specific periods to mitigate contamination. However, these traditional methods have proven insufficient. We therefore explored advanced techniques for detecting and quantifying ergot-contaminated cereals, as well as methods for reducing ergot alkaloid concentrations. **Methods**: Searches were conducted in scientific databases including Google Scholar, PubMed, and Scopus to identify research articles, reviews, and experimental studies published mainly between 2012 and August 2025, including accepted or in-press manuscripts, with special attention to works from 2021 onward to capture the most recent advancements. **Results/Conclusions**: Ultra-high-performance liquid chromatography–tandem mass spectrometry (UHPLC-MS/MS) is the reference method for confirmatory, epimer-aware quantification of ergot alkaloids, and is already standardized. Recent QuEChERS-UHPLC-MS/MS workflows in cereal matrices, including oat-based products, routinely achieve limits of quantification of about 0.5–1.0 µg/kg with single-run analysis times of about 5–15 min. Rapid screening options complement, rather than replace, confirmatory mass spectrometry: magnetic bead-based immunoassays that use magnetic separation and a smartphone-linked potentiostat provide sub-hour turnaround and field portability for trained quality-assurance staff, although external validation and calibration traceable to LC-MS/MS remain prerequisites for routine use. In practice, operators are adopting tiered, orthogonal workflows (e.g., immunoassay or electronic-nose triage at intake followed by DNA-based checks on grain washings and LC–MS/MS confirmation, or hydrazinolysis “sum parameter” screening followed by targeted MS speciation). Such combinations reduce turnaround time while preserving analytical rigor. Biotechnology also offers potential solutions for reducing ergot alkaloid concentrations at the source. Finally, to enhance consumer safety, artificial intelligence and blockchain-based food traceability appear highly effective. These systems can connect all stakeholders from producers to consumers, allowing for real-time updates on food safety and rapid responses to contamination issues. This review primarily synthesizes advances in analytical detection of ergot alkaloids, while mitigation strategies and supply chain traceability are covered concisely as supporting context for decision making.

## 1. Introduction

Ergot is a fungal disease affecting a variety of wild and cultivated cereal crops. It is caused mainly by species of the genus *Claviceps* (especially *Claviceps purpurea*), but other fungi such as *Aspergillus* and *Penicillium* have also been reported to produce ergot-like alkaloids. The infection replaces the normal grain with hardened structures called sclerotia. These sclerotia are rich in ergot alkaloids—biologically active compounds historically associated with toxicity in humans and animals when contaminated grain is consumed. Ergot poisoning (ergotism) has been linked to significant historical events and outbreaks. For example, in the Middle Ages, epidemics of St. Anthony’s Fire caused convulsions, hallucinations, and gangrene in communities that consumed ergot-contaminated rye. Some scholars have even proposed that the “dancing plagues” of the 14th–17th centuries, where people experienced uncontrollable dancing and convulsions, and the symptoms reported during the Salem witch trials of 1692, might have been related to ergotism [1,2].

Ergot alkaloids also have a fascinating connection to ancient Greek culture. The *Kykeon*, an essential sacramental drink in the Eleusinian Mysteries, is thought to have contained ergot-derived alkaloids. Consumed during secret religious rites honoring the goddesses Demeter and Persephone, the Kykeon may have induced transformative spiritual experiences with its hallucinogenic properties [3]. This idea is supported by modern research: Albert Hofmann, R. Gordon Wasson, and Carl A. P. Ruck theorized that ergot-infected barley used in the Kykeon was responsible for its psychoactive effects [4]. Their work, as detailed in *The Road to Eleusis*, connects these ancient practices with the modern understanding of psychedelics, highlighting the enduring influence of ergot on human history and spirituality.

**Figure 1:**
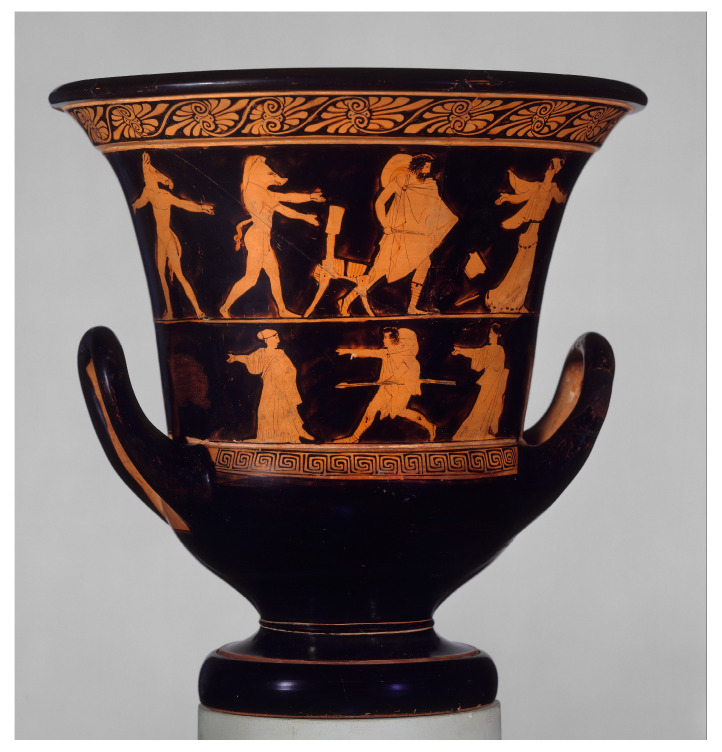
Terracotta calyx-krater. Above, Odysseus pursuing Circe; below, man between women. ca. 440 BCE. Courtesy of The Metropolitan Museum of Art, from https://commons.wikimedia.org/wiki/File:Terracotta_calyxkrater_(bowl_for_mixing_wine_and_water)_MET_DT2982.jpg (accessed on 16 November 2025), licensed under [CC BY 4.0] (https://creativecommons.org/licenses/by/4.0/).

The mythology surrounding ergot extends to literary sources as well. In Homer’s *Odyssey*, the enchantress Circe uses a potion “to turn Odysseus’s men into swines”. Odysseus resists this spell with the help of a sacred herb called “moly”. Some historians and pharmacologists interpret this episode as an allegory for toxic or hallucinogenic substances; the mythical “moly” could have been inspired by “antidotes” like *Galanthus nivalis* that counteract neurotoxic effects. The transformative peril faced by Odysseus’s crew echoes the loss of humanity and control seen in ergot poisoning [5]. This mythological context illustrates how deeply ergot’s psychoactive and toxic properties are ingrained in cultural memory (Figure 1).

Modern public health concerns about ergot contamination have prompted regulatory action. The European Commission issued Regulation (EU) 2023/915 in response to the recognized risk of ergot alkaloids in the food supply, significantly lowering the maximum allowable levels of these toxins in cereals [6]. Despite such measures, ergot contamination of grains is an enduring problem that has preoccupied humanity for centuries. The psychoactive and toxic effects associated with ergot alkaloids (convulsions, hallucinations, vasoconstriction leading to gangrene) underscore the importance of controlling this contamination. Foodborne human ergotism is rare, and we found no peer-reviewed outbreak reports in recent years; media reports from Africa lack peer-reviewed confirmation and are therefore not cited. Current risk relevance is instead evidenced by regulatory alerts of elevated ergot alkaloids in cereals and by recurring veterinary toxicoses, while most recent human case reports involve iatrogenic exposure to ergot derivatives rather than contaminated foods. For a comprehensive synthesis that links the cultural history of ergot with its fungal biology, alkaloid biosynthesis, and modern biotechnological applications, see Haarmann et al. [3].

Ergot infection of plants is favored by certain environmental conditions: high humidity, temperature fluctuations, adequate oxygen, and a susceptible host during flowering. The disease typically manifests as black or dark purple sclerotia (often called *Secale cornutum* when occurring on rye, *Secale cereale*), but contamination is not always obvious. Ergots can be small, hidden within harvested grain, or break into powder and spread, making them difficult to detect by visual inspection or taste [7]. Notably, *Claviceps* infection can sometimes act as a form of “chemical defense” for the host plant; the alkaloids deter herbivores and pathogens, essentially making ergot alkaloids a toxic by-product that protects the plant [8]. This dual nature—plant defense versus food contaminant—adds complexity to managing ergot. In summary, ergot alkaloids present a significant food safety challenge, requiring modern detection and mitigation strategies alongside historical knowledge and regulatory oversight.

**Figure 2:**
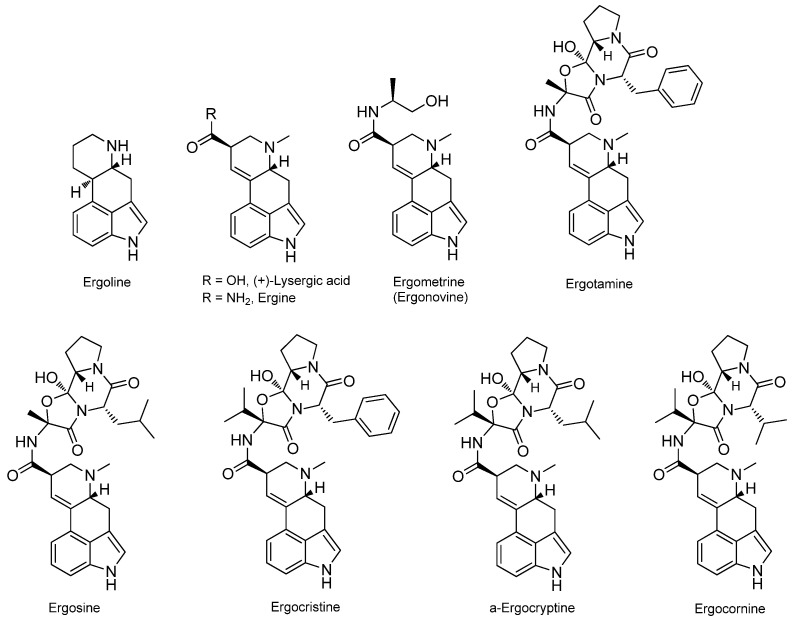
Main representatives of ergot alkaloids.

Ergot alkaloids (EA) belong to the group of indole alkaloids and are derivatives of lysergic acid [8]. More than 40 biologically active compounds have been identified in ergot, but ergotamine and ergometrine are among the most significant ones due to their established medical application (Figure 2). These alkaloids are found in varying concentrations in contaminated foods, with ergotamine often being the most prevalent among them. Elevated concentrations of ergometrine, ergosine, and ergotamine have been detected in wheat and barley samples [7]. In rye products, ergocristine and ergotamine were identified, while ergosine was the most prevalent compound in several cereal samples [9]. Additionally, ergosine, ergotamine, ergocristine, and ergocryptine were found in dairy feed [10]. In freshly prepared pasta, ergocristine, and ergotamine were the predominant ergot alkaloids, whereas in freshly cooked pasta, ergocristine was the most prevalent. This suggests that processing affects the relative concentration of ergot alkaloids [11].

This review primarily focuses on advances in analytical detection of ergot alkaloids, while mitigation approaches and supply chain traceability are treated briefly as supporting context for risk management and regulatory decision making.

Because screening and confirmation only reduce risk when results drive rapid, targeted actions, we also preview enabling digital tools, lot-level traceability, event logging, and analytics, which are treated in Section 4.1 as the operational link between laboratory findings and supply chain decisions.

## 2. Methods

To understand the current landscape of ergot alkaloid contamination and management, a comprehensive literature review was conducted. Regulatory documents, including the EU legislative actions, were examined to determine current permissible limits for ergot alkaloids in cereals and food products. Scientific databases such as Google Scholar, PubMed, and Scopus were searched for research articles, reviews, and experimental studies published primarily from 2012 to 31 August 2025, with particular emphasis on publications from 2021 onward reflecting the most recent developments. A wide range of keywords were employed during database searches:

“Mycotoxins”; “Ergot alkaloids”; “*Claviceps purpurea*”; “Lysergic acid”; “Food safety” “ergot alkaloids in food”; “Claviceps purpurea contamination”; “ergot alkaloid detection methods”; “fungal toxins cereals”; “mycotoxin mitigation strategies”; “blockchain food safety”; “AI machine learning ergot alkaloids detection”.

Initial searches yielded approximately 450 articles. We applied the following inclusion criteria:

- Peer-reviewed journal articles, regulatory reports, and validated reviews.
- Studies addressing detection, quantification, occurrence, mitigation, and management of ergot alkaloids.
- Studies presenting novel analytical, biotechnological, or digital tools relevant to food safety.

Exclusion criteria:.

- Articles unrelated to cereals or food/feed safety (e.g., pharmaceutical ergot alkaloid use).
- Studies focusing only on unrelated mycotoxins without ergot alkaloid reference.

After title/abstract screening and full-text evaluation, 33 articles were selected for detailed review and data extraction.

We appraised study quality by domain: analytical methods required full validation, including LOD/LOQ (Limit of Detection/Limit of Quantification), recoveries, precision, matrix effects and their mitigation, epimer resolution and epimerization control, matrix-matched calibration with isotope-labeled internal standards where available, measurement uncertainty, and routine QC (Quality Control) or proficiency; occurrence surveys required a defined sampling frame and sample size, LOQs suitable for comparison with applicable maximum levels, and explicit handling of left-censored data; mitigation studies required controlled designs with appropriate replication and statistical analysis; genetic or biotechnological studies required clear target and construct description with phenotype evidence and multi-environment or mechanistic support; molecular detection studies required analytical sensitivity and specificity with correlation to chemical measurements where available; digital or AI tools required an independent test set or external validation with reporting on calibration and generalizability.

This is a narrative review that integrates findings from primary research articles, regulatory documents, and technological development reports to provide a comprehensive and up-to-date overview of current challenges and solutions related to ergot alkaloid detection, mitigation, and traceability in cereals. The primary aim is to explore and integrate current innovations in ergot alkaloid detection, mitigation traceability and future challenges in the detection of such alkaloids in cereals.

## 3. State of the Art: Evidence Synthesis

### 3.1. Recent Research on Ergot Alkaloid Levels in Food: An Overview of Concentration Trends

Detecting and quantifying the prevalence of ergot alkaloids in food and feed is crucial for understanding the scope of the problem and the effectiveness of mitigation strategies. Surveys in various regions have measured ergot alkaloid levels in cereals and feed, revealing that contamination is sporadic but can reach significant levels under conducive conditions.

As summarized in Table 1, recent surveys show low to moderate ergot alkaloid occurrence with strong matrix and regional effects: in Algeria, 20% of cereal samples were positive, with wheat 26.7% at 3.66–76 µg/kg and barley 13.3% at 17.8–53.9 µg/kg, with higher levels in humid coastal areas [12]; in Thailand, 50% of 200 feed samples contained ergot alkaloids, with higher prevalence and concentrations in dairy feed, 5.9–158.7 µg/kg, dominated by ergocryptine, ergosine, and ergotamine [10]. Overall, the available data suggest that while average ergot alkaloid levels in cereals and feeds typically remain under legal limits, significant variability exists due to environmental factors, cereal species, agronomic practices, and regional differences. Climate instability, particularly increasing rainfall variability and humidity during critical flowering periods, could exacerbate the incidence and severity of future ergot outbreaks. Additionally, even when mean contamination levels are low, isolated “hot spots” of high ergot alkaloid accumulation pose acute risks to food and feed safety. Therefore, continued surveillance, combined with enhanced detection methods and mitigation strategies, remains critical in the post-2021 regulatory environment.

### 3.2. Detection of Ergot Alkaloids: Analytical Methods and Emerging Techniques

Detecting ergot contamination in cereal grains (and related products) is challenging, in part because the problem is often not visibly apparent. While large, whole ergot sclerotia can be removed by visual inspection or mechanical sorters, ergot alkaloids may also be present in fragments or dust, or even in apparently normal-looking grain. Therefore, sensitive analytical methods are needed to identify and quantify ergot alkaloids. Historically, straightforward approaches like visual inspection or simple bioassays were used, but these can fail when contamination is subtle. In recent years, considerable effort has been devoted to developing and refining detection techniques. Below, we summarize established and innovative methods for ergot alkaloid detection, highlighting their principles, advantages, and limitations.

#### 3.2.1. HPLC vs. ELISA for Ergot Alkaloid Detection

High-Performance Liquid Chromatography (HPLC) remains the cornerstone for ergot alkaloid analysis due to its accuracy, reliability, and versatility [8,10,13]. HPLC typically involves chromatographic separation of alkaloids followed by detection with ultraviolet (UV) or fluorescence detectors [8]. In many modern applications, HPLC is coupled with mass spectrometry (LC-MS) to enhance sensitivity and specificity [10,13]. Due to its robust performance characteristics, HPLC is widely regarded as the reference method against which newer, faster detection methods are evaluated.

A notable comparative study evaluated HPLC versus Enzyme-Linked Immunosorbent Assay (ELISA) for the detection of ergot alkaloids in 372 winter rye samples. These samples, derived from three rye genotypes intentionally inoculated with *Claviceps purpurea*, were analyzed using both methods [9]. The HPLC approach successfully quantified 12 common ergot alkaloids: ergometrine, ergometrinine, ergosine, ergosinine, ergotamine, ergotaminine, ergocornine, ergocorninine, α-ergocryptine, α-ergocryptinine, ergocristine, and ergocristinine. However, it failed to detect lysergic acid itself—a degradation product and biosynthetic precursor in the ergot family—likely due to its chemical instability during sample handling [8].

In contrast, the ELISA technique, using antibodies with cross-reactivity to various ergot alkaloid structures, often reported higher total alkaloid concentrations than HPLC. This higher sensitivity may result from the ELISA’s ability to detect not only intact alkaloids but also metabolites and degradation products such as lysergic acid, thereby potentially overestimating the total alkaloid burden [9]. The study concluded that while ELISA offers a useful rapid screening tool, especially in contexts like plant breeding programs aiming to select low-ergot genotypes, its quantification accuracy does not yet match that of HPLC. In regulatory and food safety contexts, ELISA results should therefore be confirmed with HPLC or LC-MS methods [9,10,13].

Ultimately, HPLC remains indispensable for precise quantification of individual ergot alkaloids, whereas ELISA provides valuable speed and high-throughput screening potential, particularly for preliminary evaluations [8,9,10,13]. The choice between methods depends largely on the application: regulatory compliance testing demands chromatographic confirmation, while initial screening of large sample sets can benefit from immunoassays [9,10,13].

#### 3.2.2. Magnetic Bead-Based Immunoassay

Magnetic bead-based immunoassays represent an emerging innovation in the detection of ergot alkaloids, offering a faster and potentially more portable alternative to traditional ELISA methods. These assays leverage magnetic microbeads coated with specific binding molecules—such as Protein G coupled with antigens or antibodies—that selectively capture ergot alkaloids from sample matrices [14].

In a recent study, Höfs et al. (2023) developed a competitive magnetic bead immunoassay specifically targeting ergometrine in rye flour [14]. The magnetic beads were functionalized with a lysergic acid–protein conjugate, mimicking the molecular structure of ergometrine. During the assay procedure, a sample extract containing unknown amounts of ergometrine competed with the coated beads for binding to free anti-ergot alkaloid antibodies. Following a brief incubation, magnetic separation enabled rapid isolation of the bead-bound complexes.

Quantification was achieved by attaching either a detection enzyme or an electrochemical tag to the antibodies, allowing the amount of ergometrine in the sample to be measured via amperometric signals. A significant advancement in this study was the integration of the assay with a portable potentiostat capable of Bluetooth communication with smartphones, thereby facilitating immediate, user-friendly readout of results.

Compared to conventional ELISA, this magnetic bead assay exhibited several key advantages:

- It eliminated the need for overnight incubations and extensive plate-coating procedures;
- It significantly reduced total assay time (to less than one hour);
- It allowed for portable, field-deployable analysis using handheld devices;
- It maintained sensitivity sufficient for detecting ergometrine levels relevant to regulatory thresholds.

In practical terms, the magnetic bead-based immunoassay demonstrated the potential for decentralized, on-site testing of cereal products, providing rapid decision-making capabilities in agricultural and food processing contexts. Although still in the validation stage, and not yet commercially available for consumers, this technology hints at a future where farmers, grain buyers, and food processors could routinely perform rapid ergot alkaloid assessments using smartphone-linked devices. Analogous to how handheld glucose meters transformed diabetic care, magnetic bead immunoassay platforms could similarly democratize food safety monitoring. The competitive magnetic bead immunoassay employs simple magnetic separation and a handheld potentiostat with smartphone readout, which enables sub-hour analyses outside a centralized laboratory. In the near term, the intended users are trained QA staff at elevators or mills, not consumers, because routine deployment still requires validated extraction, matrix-matched calibration traceable to LC-MS/MS, and external inter-laboratory verification.

Despite their portability, current magnetic bead immunoassays are not ready for consumer self-testing. Practical hurdles include:

- Solvent extraction and safe handling, typical cereal workflows use organic solvents that require trained users and fume control.
- Matrix effects and epimerization control, cereal extracts can alter antibody binding and R/S epimer ratios unless pH, light, and temperature are controlled.
- Antibody specificity and cross-reactivity, risk of false positives or negatives without well-characterized affinity profiles.
- Calibration traceability, quantitative results must be traceable to LC-MS/MS with matrix-matched calibration and, where possible, isotope-labeled internal standards used for method alignment.
- Analytical robustness, need for defined limits of detection and quantification, hook-effect checks at high concentrations, and built-in positive/negative controls.
- Stability and shelf life, bead conjugate and reagent stability across storage and field conditions.
- External validation, inter-laboratory studies and formal method validation before regulatory or buyer acceptance.

In the short term, these devices are best positioned for trained QA staff at elevators or mills, with LC-MS/MS used for confirmation and periodic method alignment.

#### 3.2.3. Microwave- and Ultrasound-Assisted Extraction Methods

Efficient extraction of ergot alkaloids from complex matrices such as grains or feed is a critical prerequisite for accurate detection. Traditional extraction methods, often involving prolonged solvent soaking or reflux, can be time-consuming and solvent-intensive. Consequently, innovative extraction techniques such as Microwave-Assisted Extraction (MAE) and Ultrasound-Assisted Extraction (UAE) have been explored for their potential to improve recovery, speed, and sustainability.

Nowak et al. (2016) investigated the applicability of MAE and UAE in the extraction of ergot alkaloids from seeds of Ipomoea species (morning glory plants), which naturally produce psychoactive ergot-related alkaloids [15]. Their study sought to determine whether these rapid techniques could efficiently extract compounds like ergometrine and lysergic acid amide for subsequent liquid chromatographic analysis.

The findings were instructive. Microwave-assisted extraction, although rapid, was deemed unsuitable for ergot alkaloid recovery. The intense localized heating generated during MAE led to chemical degradation of sensitive alkaloids, causing artificial increases in lysergic acid concentrations. This spurious elevation suggested that MAE may break down larger alkaloid molecules into lysergic acid derivatives, thus misrepresenting the true alkaloid profile and compromising analytical accuracy.

In contrast, ultrasound-assisted extraction, particularly using an ultrasonic bath (UAE-B), demonstrated excellent performance. UAE disrupted cell walls through cavitation effects, liberating intracellular alkaloids into the extraction solvent without significant degradation. The ultrasound-based method achieved good recovery rates, preserved chemical integrity, and drastically reduced extraction time compared to conventional techniques. Additionally, UAE required smaller sample and solvent volumes, supporting its adoption as an eco-friendly and efficient extraction approach for ergot alkaloids.

Based on these results, UAE has emerged as a viable and validated technique for extracting ergot alkaloids from plant and grain matrices, especially when followed by sensitive detection methods such as LC-MS/MS. By contrast, MAE should be avoided in ergot alkaloid workflows due to its propensity for analyte degradation. Optimization of UAE parameters (such as sonication time, solvent choice, and power settings) can further enhance recovery efficiencies, paving the way for faster and greener analytical pipelines.

#### 3.2.4. Hydrazinolysis for Total Ergot Alkaloid Screening

One analytical challenge in ergot alkaloid detection lies in the structural diversity of these compounds. Classical methods such as HPLC-MS/MS focus on quantifying individual alkaloids separately, necessitating multiple standards, calibration curves, and time-intensive analyses. To streamline this process, recent research has explored chemical strategies to collapse the diversity of ergot alkaloids into a single measurable entity, thereby enabling a “sum parameter” approach for total alkaloid estimation.

Hydrazinolysis, a chemical cleavage method employing hydrazine reagents, has shown promise in this context. Kuner et al. (2021) developed a novel hydrazinolysis protocol aimed at converting complex ergot peptide alkaloids into a uniform lysergic acid hydrazide derivative, which can then be quantified as a proxy for total ergot alkaloid content [16].

In their study, two cleavage pathways were compared: acidic hydrolysis followed by esterification versus hydrazinolysis. Acidic treatment was found to be suboptimal due to long reaction times, inconsistent yields, and susceptibility to oxidation artifacts, complicating the accurate measurement of total ergot content. In contrast, hydrazinolysis achieved efficient cleavage of the peptide bonds within major ergopeptine alkaloids under milder conditions and within practical reaction times (40–60 min). The addition of ammonium iodide as a catalyst further accelerated the reaction.

The hydrazinolysis approach successfully converted complex ergopeptines—such as ergotamine, ergocristine, and ergocryptine—into a uniform detectable species. Interestingly, simpler alkaloids such as ergometrine and ergometrinine, which lack the characteristic tripeptide moiety, were largely unaffected by the treatment, highlighting a limitation in total recovery. Nonetheless, for the purposes of rapid screening for major toxic ergot compounds, the hydrazinolysis method demonstrated strong potential.

Adopting such a sum parameter assay offers practical advantages:

- It reduces analytical complexity by allowing for a single quantification step instead of multi-analyte separations.
- It facilitates rapid triage of large numbers of samples in surveillance programs.
- It enables early identification of batches requiring more detailed confirmatory analysis.

Although further validation and standardization are needed—particularly regarding food matrices and regulatory thresholds—hydrazinolysis represents a promising direction for simplifying ergot alkaloid monitoring. Screening methods based on chemical derivatization could substantially enhance throughput and lower testing costs in the cereal industry.

#### 3.2.5. UHPLC-MS/MS for Multi-Alkaloid Analysis

Ultra-High-Performance Liquid Chromatography coupled with Tandem Mass Spectrometry (UHPLC-MS/MS) has emerged as the gold standard for the simultaneous detection and quantification of multiple ergot alkaloids within a single analytical run. This approach combines the advantages of rapid chromatographic separation offered by UHPLC with the sensitivity and specificity of mass spectrometry, addressing the complexity of analyzing structurally related ergot alkaloids and their epimers.

The use of UHPLC-MS/MS allows for the monitoring of multiple ergot alkaloids—typically including the 12 major compounds and their corresponding epimers—with high precision. Multiple reaction monitoring (MRM) modes are employed, in which precursor and product ion transitions are selected for each analyte, ensuring highly selective and sensitive detection even in complex food or feed matrices.

Poapolathep et al. (2021) demonstrated the application of UHPLC-MS/MS for analyzing ergot alkaloids in 200 feed samples from dairy and swine industries in Thailand [10]. The validated method could detect and quantify ergocryptine, ergosine, ergotamine, ergocornine, ergometrine, and their epimers, achieving detection limits well below regulatory maximums. The method adhered to the European Union’s SANTE/11813/2017 guidelines for mycotoxin analysis, confirming its applicability for official control purposes [17].

Similarly, Carbonell-Rozas et al. utilized UHPLC-MS/MS coupled with a QuEChERS (Quick, Easy, Cheap, Effective, Rugged, and Safe) sample preparation method to detect ergot alkaloids in oat-based foods, barley and wheat [13]. They reported low detection limits in the microgram per kilogram (µg/kg) range, along with excellent recovery rates and reproducibility, demonstrating that UHPLC-MS/MS is not only applicable to raw cereals but also to processed food products.

An important feature of UHPLC-MS/MS is its ability to quantify both *R*- and *S*-epimers of ergot alkaloids separately. Since *R*-epimers are typically more biologically active and toxic, while *S*-epimers are considered less potent, distinguishing between these forms is critical for accurate risk assessment. UHPLC-MS/MS thus supports both total alkaloid quantification and detailed toxicological profiling.

A recent QuEChERS–LC-MS/MS method in cereal-based foods, validated for the 12 ergot alkaloids together with tropane and pyrrolizidine alkaloids, reported recoveries of 71–119%, precision below 19%, and LOQs of 0.5–1.0 µg/kg, confirming the feasibility of multi-class, low-µg/kg quantification in routine workflow [18].

The primary limitations of UHPLC-MS/MS lie in its cost and technical requirements. Instruments are expensive to acquire and maintain, require highly trained personnel to operate, and necessitate rigorous method development and validation. Nonetheless, for regulatory agencies, major food producers, and research institutions, UHPLC-MS/MS remains an indispensable tool for ensuring compliance with stringent food safety standards.

Confirmatory quantification of ergot alkaloids in cereals is best achieved with epimer-resolving UHPLC-MS/MS methods, which are now harmonized for cereals and cereal products in EN 17425:2021. The recent QuEChERS–LC-MS/MS validations in cereal-based foods confirm routine LOQs of about 0.5–1.0 µg/kg for the 12 regulated alkaloids demonstrate the feasibility of multi-class panels that include tropane and pyrrolizidine alkaloids in a single run. Screening immunoassays remain useful for intake triage, yet cross-reactivity and poor agreement with chromatographic totals in rye highlight the need for LC-MS/MS confirmation before compliance decisions [9,18,19].

In summary, UHPLC-MS/MS provides unmatched analytical performance for ergot alkaloid detection, enabling precise, sensitive, and high-throughput analysis essential for both regulatory surveillance and research applications.

#### 3.2.6. Molecular Detection Methods (Loop-Mediated Isothermal Amplification-LAMP and PCR)

Most detection methods for ergot alkaloids focus on the chemicals (the alkaloids) themselves. An alternative approach is to detect the presence of the *Claviceps* fungus (or its DNA) in grain samples, since the fungus is the source of the alkaloids. Molecular biology techniques can be faster and may detect contamination even when alkaloid levels are very low. One such method is Loop-Mediated Isothermal Amplification (LAMP), a DNA amplification technique that can amplify target DNA at a constant temperature (unlike PCR which requires thermal cycling). LAMP is known for its rapidity and the possibility of being used in field settings (even colorimetric LAMP assays exist that change color when positive, requiring minimal equipment).

Researchers have explored LAMP and other DNA-based assays to identify *Claviceps purpurea* contamination. For example, Comte et al. (2017) developed quantitative PCR assays on grain washings that correlated well with actual ergot contamination levels [20]. Although that study used a standard qPCR, it demonstrated the principle that DNA from *C. purpurea* on grains can be quantified to estimate ergot contamination. A LAMP assay could similarly target a gene specific to *Claviceps* and yield a rapid yes/no (or even quantitative) result indicating if a batch of grain has fungal contamination. The advantage of such molecular methods is that they could detect the pathogen before significant toxin production occurs or in cases where toxins have leached out or degraded (e.g., in older samples).

However, there are notable limitations to molecular detection. First, finding fungal DNA does not always equate to high toxin levels—there could be DNA from spores or a very light infection that wouldn’t have produced much alkaloid. Conversely, alkaloids might persist even if DNA is degraded (for instance, in processed flour the fungus might be dead but alkaloids remain). LAMP and PCR also require careful design to avoid false positives/negatives and typically need some laboratory capability (even if simpler than full chemical analysis). In practice, these methods have not seen widespread adoption for ergot, likely because chemical analysis is quite advanced and directly measures the toxins of interest. That said, as DNA sequencing and isothermal amplification technologies advance, they could complement chemical methods—for instance, a grain elevator might do a quick DNA swab test for *Claviceps* to decide if a load should be tested more rigorously for alkaloids.

In summary, the arsenal of detection techniques for ergot alkaloids spans classical analytical chemistry (HPLC, LC-MS), immunoassays (ELISA, magnetic beads), novel extraction and processing methods (to enable faster analysis), and molecular biology tools. Each has a role: HPLC-MS/MS for confirmatory and comprehensive analysis; ELISA or magnetic assays for rapid screening; advanced extraction for speeding up workflows; and possibly DNA-based tests for early detection of contamination.

Table 2 provides a comparison of these methods and their suitability.

As summarized in Table 2, no single method fits every decision point, so operators increasingly integrate complementary tools. To shorten time to decision while maintaining confirmatory rigor, operators use orthogonal, tiered combinations. Typical pairings include intake immunoassay screening with confirmatory LC-MS/MS for presumptive positives; DNA-based assays on grain washings to trigger chemical testing when fungal presence is detected; hydrazinolysis sum-parameter screening of ergopeptines followed by targeted multiple reaction monitoring for speciation and epimers; and electronic nose triage to prioritize LC-MS/MS batches. These parallel triage plus confirmation workflows reduce turnaround and laboratory load without compromising data quality.

These tiered analytical workflows are most effective when coupled to lot-level traceability that records sampling, screening, confirmations, and release decisions so that presumptive positives trigger targeted containment and recall steps described in Section 4.1.

### 3.3. Genetic and Biotechnological Strategies to Reduce Ergot Alkaloid Contamination

Mitigating ergot alkaloid contamination at its source—before grains enter the food supply—is an appealing strategy. If we can prevent or reduce the production of ergot alkaloids in the field, the burden on post-harvest sorting and chemical analysis would lessen. Modern biotechnology offers some potential approaches, primarily through genetic modification of the fungus or the host plant to disrupt the ergot lifecycle or toxin biosynthesis.

At-source biotechnological approaches target both the pathogen and the host. On the pathogen side, strategies seek to suppress the ergot-alkaloid biosynthetic pathway by disrupting or silencing genes within the cluster, or by altering higher-order chromosomal contexts that control pathway expression. Proof-of-concept work in related fungal systems shows that reconfiguring chromosome ends can shift or ablate alkaloid production, and that keeping key pathway genes transcriptionally silent prevents toxin accumulation, which points to practical regulatory nodes for intervention [23,24]. On the host side, resistance loci associated with reduced sclerotia formation have been mapped in wheat, for example, in the durum cultivar Greenshank, which enables marker-assisted introgression and stacking in elite backgrounds [25]. Breeding programs are pairing these loci with phenology traits that shorten the infection window, for example, tighter floret closure and synchronized anthesis, to limit pathogen ingress. Endophyte and microbiome-informed strategies, including selection or introduction of benign competitors at stigma or floret surfaces, are under evaluation and may complement genetic resistance, although they require multi-environment validation. Field deployment of edited or transgenic materials, and any pathogen-targeted interventions, will require careful ecological risk assessment, consistent performance across seasons and environments, and alignment with jurisdictional regulatory frameworks.

Breeding for host resistance is progressing in wheat. In the durum cultivar Greenshank, quantitative trait loci associated with reduced sclerotia number and mass were mapped, including a locus on chromosome 5A, and lines carrying favorable alleles showed markedly lower ergot severity under inoculation. These loci can be tracked with markers for introgression into elite backgrounds, first through marker-assisted selection, then through pyramiding to increase durability. Breeding programs are combining these loci with traits that shorten the window of floral susceptibility, for example, tighter floret closure and synchronized anthesis, to reduce infection opportunities. Multi-environment validation is essential because expression varies with temperature and humidity during flowering. In practice, resistance stacking is paired with agronomic measures, for example, weed host control and targeted fungicide timing, to suppress both sclerotia formation and inoculum carryover [25].

One line of research focuses on genetically engineering the ergot-producing fungi themselves. Ergot alkaloids are synthesized by *Claviceps* (and related fungi) via a cluster of genes, many of which reside in subtelomeric (chromosome-end) regions of the fungal genome. Florea et al. used a genetic modification approach on a related fungus, *Epichloë coenophiala*, which is an endophytic fungus in grasses that also produces ergot alkaloids (in the context of tall fescue toxicosis) [24]. They introduced a specific gene into *Epichloë* aimed at knocking out the expression of the toxin-production genes. In essence, this added gene acts as a molecular switch to turn off the pathway responsible for ergotamine and other ergot alkaloid biosynthesis. The modified fungus showed a greatly reduced capacity to produce ergot alkaloids. Although *Epichloë* in forage grass is a different scenario than *Claviceps* in cereal heads, the concept is similar: if the pathogen could be rendered incapable of producing toxins, ergot would become a much less severe problem.

In a related study, Fabian et al. (2018) examined why some fungi do not produce ergot alkaloids under certain conditions [23]. Specifically, they looked at *Penicillium camemberti* (the fungus used in Camembert cheese rind), which is known to have the genetic potential to make ergot alkaloids but typically does not produce them during cheese fermentation [23]. By investigating gene expression in *P. camemberti*, they found that certain genes in the ergot alkaloid pathway are kept inactive, explaining the lack of toxin in cheese rinds. This understanding points to the importance of gene regulation: if the ergot alkaloid genes can be kept “turned off” (through genetic engineering or natural strain selection), contamination can be avoided. These insights from *Epichloë* and *Penicillium* research suggest that controlled gene expression in the fungi can effectively reduce or eliminate alkaloid production.

Another approach targets the plant side of the interaction. Instead of modifying the fungus, researchers are breeding or engineering cereal crops to be more resistant to *Claviceps* infection or less conducive to alkaloid accumulation. A study by Gordon et al. mapped genetic loci in a variety of durum wheat (*Triticum turgidum* cv. Greenshank) that confer resistance to ergot [25]. They identified four specific gene loci associated with resistance traits, including one on chromosome 5A (named QCp.aafc.DH-5A) linked to reduced sclerotia development. Resistant wheat plants had fewer and smaller sclerotia when infected, which directly correlates with lower ergot alkaloid levels in the grain. The study also noted that host plant hormone regulation (e.g., factors affecting flower closure or susceptibility) plays a role in how well *Claviceps* can infect and spread. One intriguing idea proposed was to develop fertility restoration genes in the plant that prevent the fungus from hijacking the plant’s reproductive process (ergot replaces the seed, so if the plant can be made to abort infected ovules or otherwise foil the fungus, it stops the cycle).

By combining these resistance genes into new cereal cultivars (via marker-assisted breeding or genetic engineering), the incidence of ergot could potentially be greatly reduced. Indeed, modified wheat lines in experiments showed significantly fewer successful infections by *C. purpurea*. In practice, they still got some infections, but often the fungus could not complete its life cycle (the pathogen might infect a floret but fail to produce a viable sclerotium). This results in dramatically lower spore production for the next cycle and reduced spread in the field.

However, there are important considerations before these genetic solutions can be widely implemented. Engineering the fungus itself (as with *Epichloë*) and then introducing it into the field has ecological and regulatory hurdles—releasing or encouraging a genetically modified pathogen (even a “benign” one) carries risks and public acceptance issues. Breeding resistant crops is less controversial, but the ergot fungus is very adaptable; there are many *Claviceps* strains, and a resistance that works in one region may be overcome in another. Additionally, any genetic modification approach must ensure that it doesn’t inadvertently affect the crop yield or quality, and that it remains effective over multiple seasons. Concerns about transgenic approaches include the possibility of the modified traits spreading to other organisms or the environment in unintended ways.

For example, if a gene to suppress alkaloid production in the fungus or the plant affects symbiotic organisms or non-target species, new ecological imbalances could arise. Ensuring that these interventions remain specific, stable, and safe will be a key focus of future research and regulatory scrutiny.

In summary, genetic and biotechnological interventions—either by disarming the fungus or arming the plant—are promising strategies to reduce ergot alkaloid contamination at the very source. Florea et al. (2016) and Fabian et al. (2018) demonstrated that silencing toxin genes in fungi is feasible, and Gordon et al. (2020) showed that enhancing host resistance can dramatically cut down ergot formation [23,24,25]. These approaches could, in theory, provide long-term solutions to ergot, reducing reliance on chemical fungicides or extensive post-harvest cleaning. However, practical deployment is still in early stages. Economic factors (development cost, time to market for new crop varieties) and ecological concerns need to be addressed. The balance of benefits (safer crops, less toxin) versus risks (potential unintended effects of genetic modifications) will determine how widely these strategies are adopted. Table 3 summarizes these genetic and biotechnological strategies aimed at reducing ergot alkaloid contamination.

### 3.4. Practical Field and Post-Harvest Mitigation Strategies

While genetic and biotechnological solutions offer promising long-term strategies to reduce ergot contamination, immediate and practical measures are essential for current cereal production systems. Several agronomic, chemical, and post-harvest interventions can mitigate ergot alkaloid contamination effectively. These strategies form an integrated approach to managing the risk from field to fork.

#### 3.4.1. Use of Fungicides

Targeted application of fungicides during critical crop development stages can suppress *Claviceps purpurea* infection. Alaoufi et al. (2023) evaluated four fungicide pre-mixes applied around wheat flowering: azoxystrobin + propiconazole, fluxapyroxad + pyraclostrobin, pydiflumetofen + propiconazole, and metconazole + prothioconazole [27]. The results demonstrated that all treatments reduced sclerotia formation and ergot alkaloid levels to varying degrees, with pydiflumetofen + propiconazole showing the most pronounced effect.

Fungicides likely act by reducing fungal colonization during the narrow window of floral susceptibility. However, complete eradication of ergot infection was not achieved, underlining the limitations of chemical protection. Moreover, fungicide application timing, persistence, and mode of action compatibility with *Claviceps* biology are critical factors influencing efficacy. While fungicides can be integrated into ergot management programs, they must be applied judiciously to minimize costs, environmental impact, and the risk of developing resistance.

#### 3.4.2. Ammoniation of Contaminated Grain

Ammoniation has been successfully employed to detoxify certain mycotoxins, notably aflatoxins, and is now being investigated for ergot alkaloids. Cherewyk et al. (2022) demonstrated that treating contaminated grains with ammonia under controlled conditions induced chemical modifications in ergot alkaloid structures, notably shifting toxic *R*-epimers toward less toxic *S*-epimers [26].

This chemical transformation reduces the biological activity of the alkaloids, rendering the grain safer for animal feed use. However, several considerations must be addressed before widespread application:

- Validation that total toxicity is significantly lowered, not merely altered.
- Assurance of grain nutritional quality post-treatment.
- Regulatory approvals regarding chemical treatments in feed and potential residues.
- Economic feasibility for large-scale application.

While ammoniation shows great potential for salvaging moderately contaminated grain lots, its use is likely to be limited to feed rather than human food applications, pending further safety evaluations.

#### 3.4.3. Food Processing Effects

Food processing steps such as cleaning, milling, and thermal treatments influence ergot alkaloid content in final products. Tittlemier et al. (2019) showed that milling durum wheat into semolina effectively reduced alkaloid concentrations by segregating sclerotia and contaminated fractions into bran and screenings [11]. However, cooking processes, such as pasta boiling, induced minimal alkaloid degradation but did cause epimerization from *R*- to *S*-forms.

Thus, while processing can redistribute or slightly alter ergot alkaloids, it cannot be relied upon for complete detoxification. The most significant reductions are achieved during physical removal stages (cleaning and milling), reinforcing the need for strict grain quality control before processing.

#### 3.4.4. Integrated Mitigation Approach

An effective ergot management system should integrate multiple strategies:

- Agronomic practices: crop rotation, timely harvesting, removal of wild grass hosts, use of less susceptible cultivars, and irrigation management to minimize conducive conditions for Claviceps infection.
- Chemical interventions: targeted fungicide applications during flowering in high-risk environments.
- Post-harvest sorting: using gravity tables, optical sorters, and mechanical cleaners to remove sclerotia and contaminated particles.
- Chemical detoxification: ammoniation for feed grains when contamination exceeds safe thresholds but remains recoverable.
- Continuous surveillance: rapid field testing and confirmatory laboratory analysis using advanced detection methods such as UHPLC-MS/MS.

By employing a holistic, multi-layered approach, cereal producers and processors can significantly reduce the incidence and severity of ergot alkaloid contamination, enhancing food and feed safety (Table 4).

## 4. Discussion

Effective management of ergot alkaloid contamination requires a comprehensive farm-to-fork strategy encompassing prevention, monitoring, detection, and mitigation. Prevention at the agricultural level—through agronomic practices, resistant cultivars, and judicious fungicide use—remains the most sustainable long-term approach. However, due to the unpredictable nature of environmental factors favoring *Claviceps purpurea* infection, complete prevention is often unattainable. Consequently, robust detection methods and post-harvest mitigation measures are vital safety nets in the cereal supply chain.

As discussed, genetic and biotechnological strategies offer exciting possibilities. Targeted modification of either the pathogen or the host plant genome to reduce ergot alkaloid production at the source represents a proactive future-oriented solution [23,24,25]. Nonetheless, widespread deployment of genetically engineered organisms must carefully balance efficacy, regulatory frameworks, ecological risk, and public acceptance.

Chemical and field interventions, such as fungicide application, ammoniation of contaminated grains, and rigorous post-harvest sorting, form the practical toolkit available today. These strategies, while effective to varying extents, underline the reality that no single measure is sufficient alone. Instead, an integrated management approach combining multiple interventions, adapted to local conditions and risk profiles, offers the best protection against ergot alkaloid contamination.

Importantly, food safety must also account for the consumer’s role. Regulatory standards, such as those set by the European Commission, continue to evolve to reflect new toxicological insights and consumption patterns. For instance, recent tightening of permissible ergot alkaloid levels reflects a precautionary approach to protect vulnerable groups like children or pregnant women, who may be more susceptible to cumulative low-dose exposures [28,29].

Technological innovations—including digital traceability systems and artificial intelligence-enhanced detection methods—are poised to dramatically improve our ability to monitor and respond to contamination events swiftly and effectively. These developments, discussed below, represent the next frontier in food safety for ergot alkaloids and mycotoxins more broadly (Box 1).

### 4.1. Compliance with EU Maximum Levels and Best Practice Pathway

Commission Regulation (EU) 2023/915 consolidates maximum levels for ergot sclerotia and for the lower-bound sum of 12 ergot alkaloids, including epimers, in specified cereal matrices. The consolidated text as of 22 July 2024 includes phased application dates for selected entries. In practice, compliance depends on three linked elements, representative intake screening, confirmatory LC-MS/MS that resolves epimers and reports the lower-bound sum, and documented decisions on cleaning, diversion, or rejection, all supported by traceability [6].

Box 1 **Best practice pathway for operators**

**Stage**

**Actions**
Know the standard and scope
Apply Commission Regulation (EU) 2023/915 for the relevant cereal or product category and measure the lower-bound sum of the 12 regulated ergot alkaloids, including *R-* and *S-* epimers.Follow official sampling and performance principles for mycotoxins to manage hotspot variability and to generate legally defensible composite samples.
Intake sampling and screening
Use risk-based sampling at intake. Perform rapid screening on the laboratory test portion, for example, immunoassay or electronic nose, to triage lots without delay.Hold presumptive positives for confirmation.
Confirmatory analysis
Confirm with UHPLC-MS/MS using a validated method that resolves epimers and reports the required lower-bound sum. EN 17425:2021 provides a harmonized approach for cereals and cereal products [19].Control epimerization during preparation, use matrix-matched calibration with isotope-labeled internal standards where available, and document uncertainty.
Decision and actions
Compliant, release the lot.Borderline, recheck sampling representativeness, repeat confirmation, consider enhanced cleaning or segregation.Non-compliant for food, do not blend to dilute for food use. Consider post-harvest cleaning or diversion to feed where permitted by feed law, or reject the lot. Document every action.
Supplier and process control
Embed agronomic requirements for ergot risk in supplier contracts and audit cleaning capability, for example, gravity tables and optical sorters.Track corrective actions and trends to reduce future non-compliance.
Traceability and records
Link lot identifiers, sampling plans, screening results, confirmatory certificates, and release decisions in a traceable digital record to enable rapid traceback and recall if required.
Laboratory quality
Maintain method validation and routine QC within accepted recovery and precision ranges, participate in proficiency testing, and keep LOQs at or below levels suitable for enforcement and surveillance.

### 4.2. Implications of Food Traceability and Consumer Intelligence Technologies

Digital technologies translate analytical findings into timely, auditable actions across the grain chain. Blockchain-style ledgers can store immutable lot movements, storage conditions, and test results to accelerate traceback and forward tracking, while analytics on these records support targeted holds and recalls and reduce time to disposition. In parallel, data-driven sensors and models, for example, portable chromatographic platforms and electronic-nose classifiers, provide rapid field triage whose outcomes should be captured in the same traceability layer and confirmed by LC-MS/MS for compliance. Together, these tools close the loop from detection to decision, improving both speed and precision of response [22,30,31].

The globalization and complexity of modern food supply chains have made rapid and reliable traceability systems essential for ensuring food safety. In the context of ergot alkaloid contamination, the ability to trace contaminated cereal lots back to their source—and forward through all stages of the supply chain—is crucial for timely interventions, recalls, and risk management. Emerging digital technologies, particularly blockchain-based traceability and consumer intelligence (CI) platforms, offer powerful tools to enhance responsiveness and transparency.

Blockchain technology, originally developed for cryptocurrency transactions, provides a decentralized, tamper-proof ledger where every transaction or event is recorded immutably. Applied to food safety, blockchain allows for the real-time recording of grain movements, quality testing results, storage conditions, and processing steps at each node of the supply chain. Singh et al. (2022) highlighted that blockchain-enabled food traceability systems can reduce the time needed to identify and isolate contaminated products from days to seconds, dramatically improving public health responses [30].

For example, if ergot alkaloid levels exceeding regulatory limits are detected in a batch of rye flour at a processing plant, blockchain records can immediately trace that batch back to its farm of origin, including all intermediate handlers. Similarly, downstream products containing the contaminated batch can be rapidly identified, enabling efficient recalls before affected products reach consumers. Such speed and accuracy would be nearly impossible with traditional paper-based or siloed digital systems.

Coupled with blockchain, consumer intelligence platforms serve as an additional layer of surveillance. These platforms allow consumers, retailers, or health authorities to report quality concerns, unusual symptoms, or suspected food safety incidents. Digital traceability and participatory surveillance translate analytical findings into timely, auditable actions. When lot-level test results are captured in interoperable traceability systems and linked to official alert and complaint channels, such as the EU Rapid Alert System for Food and Feed and U.S. FDA reporting frameworks, traceback and targeted recalls are substantially accelerated. Peer-reviewed studies on blockchain-enabled traceability and portable analytics further support these gains in responsiveness and data integrity [30,32]. In practice, this means that even subtle or geographically dispersed issues—such as minor symptoms resulting from low-level ergot alkaloid exposure—can be detected earlier than would be possible by relying solely on clinical reporting or regulatory testing. By triangulating consumer reports, blockchain data, and laboratory results, food safety agencies and industry stakeholders can adopt a more proactive posture.

Challenges remain, including ensuring universal participation across the supply chain, safeguarding data privacy, and harmonizing international standards. Nonetheless, the integration of blockchain traceability and consumer intelligence platforms represents a transformative advancement in food safety management, particularly for complex contamination threats like ergot alkaloids.

### 4.3. Future Outlook: Artificial Intelligence and Machine Learning in Ergot Detection

Recent advances in artificial intelligence (AI) and machine learning (ML) are poised to significantly enhance the detection and management of ergot alkaloids and related mycotoxins in cereals. These technologies offer promising alternatives and complements to traditional chemical analytical methods, particularly by providing rapid, cost-effective, and potentially portable solutions for contamination detection in the field.

#### 4.3.1. AI-Enhanced Chromatographic and Spectroscopic Analysis

Machine learning models have increasingly been integrated with analytical chemistry techniques, such as chromatography (HPLC, UHPLC-MS/MS) and spectroscopy (NIR, FTIR), to enhance their accuracy, speed, and efficiency. Recent studies have demonstrated that ML algorithms can interpret complex chromatographic and spectroscopic datasets more quickly and reliably than manual analysis, significantly reducing the time required for contamination detection and quantification.

Jakob et al. (2024) introduced a portable, AI-driven detection system known as “2LabsToGo,” capable of rapidly analyzing ergot alkaloids in cereals using ML algorithms to interpret chromatographic data in real-time [31]. This open-source system leverages pattern recognition and predictive modeling to classify contamination levels instantly, enabling immediate decisions on grain quality at harvest or storage points.

These approaches demonstrate how AI can not only accelerate detection but also refine data interpretation, overcoming the limitations of conventional peak-based analyses.

#### 4.3.2. Electronic Nose and Neural Network Applications

Another promising frontier involves the use of electronic noses (e-noses), devices designed to detect volatile organic compounds (VOCs) associated with fungal contamination in cereals. While ergot alkaloids themselves are non-volatile, fungal infection often generates distinct volatile chemical profiles detectable by these sensors.

A recent study by Piergiovanni et al. (2025) investigated the capability of an electronic nose to accurately predict EA contamination in wheat, presenting a proof of concept for its application [22]. When paired with supervised analytical techniques, their system achieved over 95% accuracy in differentiating clean grain from ergot-contaminated samples, highlighting the feasibility of non-destructive, real-time screening methods at grain processing facilities or even farms.

Building upon previous work by Camardo Leggieri et al. (2021) on aflatoxin and fumonisin detection in maize, this new application confirms the versatility of electronic nose systems coupled with AI models for rapid mycotoxin surveillance across different cereal species [33].

#### 4.3.3. Field-Deployable AI-Based Systems

Field-deployable AI-based systems such as “2LabsToGo” represent a notable shift toward decentralized mycotoxin analysis. These platforms typically include compact sensor units, embedded AI processing modules, and interfaces (e.g., smartphone apps) for immediate data interpretation and decision-making.

The primary advantage of these AI-enabled portable systems is the substantial reduction in turnaround time compared to laboratory-based analyses, which often require extensive sample preparation, specialized equipment, and days to weeks for results. Moreover, real-time field-based testing minimizes logistical challenges associated with sample transportation and storage.

In practical terms, such systems could allow farmers, grain handlers, and processors to screen bulk lots for ergot alkaloids at critical control points, reserving laboratory confirmation only for suspect or borderline samples.

#### 4.3.4. Advantages, Challenges, and Future Potential

The main advantages of AI-driven detection methodologies include:

- Increased analytical speed.
- Improved sensitivity and specificity.
- Cost-effectiveness for large-scale monitoring.
- Portability and suitability for field deployment.
- The ability to learn and improve continuously as more data are collected.

However, several challenges remain:

- Large, high-quality training datasets are needed to build robust models.
- Generalizability across different grain types, climates, and fungal strains must be validated.
- Long-term calibration stability must be ensured.
- Regulatory acceptance of AI-based methods requires rigorous standardization and validation processes.

Future research should prioritize enhancing model robustness, developing standardized AI protocols aligned with regulatory frameworks, and integrating AI detection tools with broader food safety management platforms such as blockchain traceability and consumer intelligence networks. As sensor technologies and computational capabilities continue to advance, AI-based solutions are expected to become indispensable components of comprehensive ergot alkaloid monitoring systems.

## 5. Conclusions

This review comprehensively examined the persistent challenge of ergot alkaloid contamination in cereals, highlighting the evolving strategies for its detection, mitigation, and prevention. While ergot alkaloids have posed a threat to human and animal health for centuries, recent scientific and technological advancements offer powerful new tools to manage this ancient problem more effectively.

Operational compliance in the EU rests on three linked pillars, measurement against the lower-bound sum of 12 ergot alkaloids including epimers under Commission Regulation (EU) 2023/915, application of the official sampling and performance principles for mycotoxins to control hotspot heterogeneity and ensure defensible lot classification, and risk framing using EFSA’s health-based guidance values. Together, these anchors define the analytical targets, evidentiary quality, and decision context for lot release, segregation, or diversion [28,29,33].

Traditional detection methods such as High-Performance Liquid Chromatography (HPLC) and Ultra-High-Performance Liquid Chromatography coupled with Tandem Mass Spectrometry (UHPLC-MS/MS) remain indispensable for precise quantification and regulatory compliance. However, the field is rapidly advancing. Novel approaches—including magnetic bead-based immunoassays, hydrazinolysis sum-parameter screening, and molecular techniques like Loop-Mediated Isothermal Amplification (LAMP)—have significantly expanded the detection toolbox, offering faster, more scalable, and sometimes field-deployable solutions.

Genetic and biotechnological interventions, including the development of ergot-resistant cereal cultivars and the targeted manipulation of fungal biosynthetic pathways, provide promising avenues for reducing contamination at its source. Nonetheless, their widespread implementation requires careful consideration of ecological impacts, regulatory approval, and public acceptance.

At the post-harvest level, practical mitigation strategies such as fungicide applications during flowering, mechanical cleaning, optical sorting, and ammoniation of contaminated grain have proven valuable in managing ergot risks. However, none of these measures is sufficient alone. A truly effective defense against ergot alkaloids requires an integrated, multi-layered approach combining prevention, detection, and remediation.

The emergence of digital technologies—particularly blockchain-based food traceability systems and consumer intelligence platforms—enhances the capacity for rapid, transparent, and coordinated responses to contamination incidents. Furthermore, artificial intelligence and machine learning are poised to revolutionize ergot detection, enabling real-time analysis, predictive surveillance, and decentralized decision-making through field-deployable systems like electronic noses and AI-enhanced chromatographic platforms.

Future research and implementation efforts should prioritize the following in rank order of anticipated impact:

- First, deploying rapid intake-screening tools integrated with digital traceability is expected to yield the most immediate risk reduction. These tools enable early identification of contaminated lots and allow for timely segregation, diversion, or recall, reducing consumer exposure.
- Second, AI-supported detection systems—such as spectral classification, pattern recognition, and e-nose platforms—show strong promise for on-site testing, but require standardization and validation against reference methods to ensure regulatory acceptance.
- Third, breeding durable resistance into cereals remains a sustainable long-term solution, especially for regions with recurrent ergot risk. Genomic tools and mapped QTLs are available, but deployment depends on multi-season trials and agronomic trade-offs.
- Fourth, improving early-warning capabilities and surveillance infrastructure—including outbreak modeling, mobile alert platforms, and enhanced data exchange between field and industry actors—will help prevent local contamination events from becoming widespread, particularly under shifting climate conditions.

Fortunately, current surveillance data suggest that most cereals and derived products comply with existing ergot alkaloid regulatory limits. However, ongoing vigilance remains essential, especially as climate variability may create conditions favoring *Claviceps* outbreaks. Continuous improvement in detection, prevention, and management systems will ensure that the risks posed by ergot alkaloids remain minimized, safeguarding public health, maintaining consumer confidence, and protecting economic interests in the global cereal industry.

In conclusion, meeting the ergot alkaloid challenge demands a dynamic, interdisciplinary strategy—one that bridges traditional agricultural practices, cutting-edge biotechnology, analytical innovation, and digital transformation. By combining these elements thoughtfully, the food safety community can continue to drive progress toward a safer, more resilient food supply chain.

Over the next 5–10 years, the largest reduction in risk will likely come from broad deployment of rapid intake screening linked to digital traceability and automated decision rules, while breeding-driven resistance delivers larger, longer-horizon gains

## Figures and Tables

**Table 1:** Recent occurrence of ergot alkaloids in cereals and feeds.

Matrix, Region, Years	n	Method	% Positive	Range, Sum EAs (µg/kg)	Dominant EAs/Notes	Lit.
Wheat, barley, Algeria, 2019–2020	60	UHPLC-MS/MS	Wheat 26.7%; barley 13.3%	Wheat 3.66–76; barley 17.8–53.9	Wheat: ergosine, ergocryptine, ergocristine; barley: ergotamine	[12]
Dairy/swine feeds, Thailand	200	UHPLC-MS/MS	50%	5.9–158.7	Ergosine, ergocryptine, ergotamine	[10]

**Table 2:** Comparison of various analytical methods for ergot alkaloid detection and their status in current practice.

Method	Status	Target Measurand	ΕR ^a^	Detection Limit/ Benchmark	Time to Result	PC ^b^	FA ^c^	Primary Strengths	Main Limitations	Typical Use Case	Lit.
ELISA (total immunoreactivity)	Screening, partially suitable	Total EA Immunoreactivity	No	Kit dependent, typically 5–20 µg/kg	2–4 h	Low	No	High-throughput, simple workflow	Cross-reactivity and bias vs. LC-MS/MS, matrix effects	Intake triage, lot prioritization	[9]
Magnetic bead-based immunoassay	Emerging, promising	Single EA target, e.g., ergometrine	No	~1 µg/kg for ergometrine, research-grade	30–60 min	Low–moderate	Yes, for trained QA	Portable, smartphone-linked readout, rapid	Needs external validation, matrix-matched calibration, epimerization control	On-site QA triage	[14]
MAE (microwave-assisted extraction)	Unsuitable (for EAs)	Extraction step, not a detector	N/A	N/A	Minutes	Moderate	No	Fast energy input	Degradation of labile EAs, spurious lysergic acid	Not recommended for EA workflows	[15]
UAE-B (ultrasonic bath extraction)	Validated as extraction	Extraction of EAs prior to analysis	N/A	Recovery typically ~85–95%	~30 min extraction	Low–moderate	No	Gentle, preserves alkaloids, reduced solvent/time	Needs matrix-specific optimization	Preferred extraction in some matrices before LC-MS/MS	[15]
Hydrazinolysis + HPLC/LC-MS	Under development	Sum-marker for ergopeptines	N/A	Indirect, sum marker not compound-specific LOQ	~2–3 h total including 40–60 min reaction	Moderate	No	Rapid total screen of major ergopeptines	Poor response for ergometrine/ergometrinine; confirmation required	High-throughput triage prior to targeted LC-MS/MS	[16,21]
Acidic esterification (cleavage)	Unsuitable	Chemical cleavage approach	N/A	N/A	>3 h	Moderate	No	—	Oxidation artifacts, inconsistent yields	Not recommended	[16]
HPLC-FLD/UV	Validated	12 EAs, partial epimers	Partial	5–20 µg/kg, matrix dependent	3–5 h including prep	Higher	No	Robust quantification where MS Unavailable	Lower specificity than MS, matrix fluorescence	Surveillance in labs without MS	[9]
UHPLC-MS/MS	Validated, advanced	12 EAs plus R/S epimers	Yes	~0.5–1.0 µg/kg typical LOQs	1–2 h total, 5–15 min per run	Moderate	No	High sensitivity/ specificity, multiplex quantification	Requires specialized instrumentation and training	Confirmatory and regulatory analysis	[10,13]
UHPLC-MS/MS with QuEChERS	Validated	12 EAs plus *R/S* epimers in cereal foods	Yes	<1 µg/kg typical	~2 h total, 5–15 min per run	Moderate	No	High throughput for cereal matrices, widely adopted	Control of epimerization needed; matrix effects possible	Routine QC/ monitoring	[13,18]
EN 17425:2021 LC-MS/MS (sum method)	Standardized	Lower-bound sum of 12 EAs including epimers	Yes	Low µg/kg, validated per standard	~1–2 h total, 10–20 min injection	Moderate	No	Harmonized method for official control	Sum-range constraints; instrumentation required	Official control/compliance	[19]
qPCR/LAMP (molecular)	Supplementary, indirect	*C.purpurea* DNA	N/A	~10 copies DNA (method dependent) ^d^	~1–2 h	Low–moderate	Yes for LAMP	Early indicator of pathogen presence; simple equipment	DNA presence ≠ toxin level; chemical confirmation required	Rapid lot triage to trigger LC-MS/MS	[20]
Electronic nose (chemometrics)	Emerging triage	VOC patterns linked to infection	N/A	Classification accuracy, not concentration; >95% in POC studies	Minutes	Very low	Yes	Rapid, non-destructive screening of bulk lots	Needs robust calibration across varieties/storage; non-regulatory	Prioritize samples for confirmatory LC-MS/MS	[22]

^a^ Epimer resolution; ^b^ Prep complexity; ^c^ Field applicability; ^d^ Molecular limits are in DNA copy numbers and are not directly comparable to µg/kg toxin concentrations.

**Table 3:** Methods for Reducing Ergot Alkaloid Concentrations in Raw Materials.

Method	Main Use/Mechanism	Key Limitations	Lit.
Biotechnology (Genetic Engineering)	Silencing or deleting genes involved in ergot alkaloid biosynthesis (e.g., in *Claviceps* or host plants)	Challenges with vector use, GMO regulation issues, ecological risks	[23,24,25]
Ammoniation of Contaminated Cereals	Chemical treatment alters epimer distribution: reduces toxic *R*-epimers, increases less toxic *S*-epimers	Potential alteration of grain nutritional quality; regulatory approval needed for food/feed	[26]
Fungicides (e.g., Pydiflumetofen + Propiconazole [PYD + PROP])	Prevents *Claviceps* infection during flowering stage, reducing initial contamination	Requires precise application timing; partial effectiveness; environmental impact concerns	[27]
Optical Sorting and Cleaning	Mechanical removal of sclerotia from grain lots	Cannot remove invisible or fragmented contamination; expensive for large-scale operations	[11]
Breeding Resistant Crop Varieties	Develop cereal lines with reduced susceptibility to ergot infection	Long breeding timelines; partial resistance only; regional strain differences	[25]

**Table 4:** Practical Mitigation Strategies Across the Food Chain.

Stage	Strategy	Notes
Pre-Harvest	Resistant cultivars, fungicide use, crop rotation	Limits initial fungal infection.
Harvest	Timely harvesting, minimizing humid conditions	Reduces risk of late-season infection.
Post-Harvest	Sorting (optical, gravity), cleaning	Removes ergot bodies from grain.
Storage	Low humidity, controlled conditions	Prevents fungal spread and alkaloid increase.
Processing	Milling, dehulling, selective flour production	Reduces contamination in food fractions.
Detoxification	Ammoniation	Chemical detoxification of contaminated grain (mostly for feed).

## Data Availability

No new data were created or analyzed in this study. Data sharing is not applicable to this article.
